# Karrierewege und Arbeitszufriedenheit in der Schmerzmedizin

**DOI:** 10.1007/s00482-024-00851-9

**Published:** 2024-12-04

**Authors:** Joachim Erlenwein, Benedikt Kube, Dirk Boujong, Joachim Nadstawek, Michael Hüppe, Tim P. Jürgens, Winfried Meißner, Frank Petzke

**Affiliations:** 1 Klinik für Anästhesiologie, Universitätsmedizin Göttingen, Robert-Koch-Str. 40, 37075 Göttingen, Deutschland; 2 Schmerztagesklinik, Stiftungskrankenhaus Nördlingen, Nördlingen, Deutschland; 3 Schmerzzentrum Bonn, Bonn, Deutschland; 4 Klinik für Anästhesiologie und Intensivmedizin, Universität zu Lübeck, Lübeck, Deutschland; 5 Neurologisches Zentrum, Klinik für Neurologie, MKG Klinikum Güstrow, Güstrow, Deutschland; 6 Klinik und Poliklinik für Neurologie, Universitätsmedizin Rostock, Rostock, Deutschland; 7 Klinik für Anästhesiologie und Intensivmedizin, Universitätsklinikum Jena, Jena, Deutschland

**Keywords:** Anästhesiologie, Arbeitszufriedenheit, Nachwuchsmangel, Gesundheitsversorgung, Schmerzpsychologie, Anesthesiology, Job satisfaction, Shortage of young professionals, Healthcare, Pain psychology

## Abstract

**Hintergrund:**

Schmerzmedizin ist eine interdisziplinäre und interprofessionelle Spezialisierung. Aufgrund von Nachwuchssorgen und Überalterung, insbesondere bei Ärzten, ist es von Bedeutung, berufliche Entwicklung und Karrierewege in die Schmerzmedizin besser zu verstehen.

**Ziel der Arbeit:**

Ziel dieser Untersuchung ist es, die beruflichen Wege von Menschen, die in einer Einrichtung der spezialisierten schmerzmedizinischen/schmerztherapeutischen Versorgung tätig sind, deren Motivation und Arbeitszufriedenheit zu erfassen.

**Material und Methoden:**

Mit einem standardisierten Online-Fragebogen wurden über kooperierende Fachgesellschaften und Verbände deren Mitglieder befragt.

**Ergebnisse:**

Es wurden Angaben von 398 Ärzten, 78 Psychologen, 62 Physiotherapeuten, 3 Ergotherapeuten und 23 Pflegefachpersonen in die Auswertung einbezogen. Die Altersverteilung lag eher in höheren Altersgruppen, der größte Anteil an Antwortenden war zwischen 51 und 60 Lebensjahren. Auf den Karrierewegen kamen die Teilnehmenden erst nach vielen Jahren Berufstätigkeit klinisch mit der Schmerzmedizin in Kontakt. Gerade bei Ärzten zeigt sich dabei eine Latenz von fast einem Jahrzehnt nach Approbation (8 ± 6 Jahre (max. 30) bzw. 5 ± 6 (min. 0, max. 28) Jahre), bzw. bereits als Facharzt gearbeitet zu haben, bis zur ersten klinischen Tätigkeit in einer spezialisierten schmerzmedizinischen Einrichtung (Psychologen 5 ± 5 (min. 0, max. 22) Jahre nach ihrem Studienabschluss bzw. 4 ± 5 (min. 0, max. 16) Jahre nach Approbation; PEP 1 ± 9 (min. 0, max. 37) Jahre nach Ausbildungsabschluss). Hinsichtlich motivationaler Faktoren werden intrinsische Aspekte höher bewertet als monetäre. Vereinbarkeitsfragen standen eher im Mittelfeld der Gewichtung. Bzgl. der Arbeitszufriedenheit in der Schmerzmedizin zeichnet sich bei den Antwortenden ein Bild mit insgesamt höheren Werten bei der Angabe der Zufriedenheit ab, wobei in den Kategorien „Aufstiegsmöglichkeiten“, „Zusatzverdienstmöglichkeiten“ und „Anerkennung der Tätigkeit bei Vorgesetzten“ die niedrigsten Zufriedenheitswerte angegeben werden. Über 1/3 der Befragten gab an, dass berufliche Änderungen mit Wechsel aus der Schmerzmedizin anstehen, insbesondere der Rentenbeginn.

**Diskussion:**

Die Ergebnisse unterstreichen die Wahrnehmung kritischer Zukunftsaussichten und lassen Risiken hinsichtlich der zukünftigen Versorgungssicherung von Menschen mit chronischen Schmerzen erkennen. Sie liefern erstmals einen Einblick in die Wege von Mitarbeitenden in die Schmerzmedizin und zu deren Motivationen und Arbeitsplatzzufriedenheit, die bei der Diskussion um die personelle Zukunftssicherung Berücksichtigung finden sollten.

**Zusatzmaterial online:**

Zusätzliche Informationen sind in der Online-Version dieses Artikels (10.1007/s00482-024-00851-9) enthalten.

## Abstract (en)

**Background:**

Pain medicine is an interdisciplinary and interprofessional field of specialisation. Due to concerns about new recruits and an aging workforce, especially among physicians, it is important to better understand professional and career pathways in pain medicine.

**Objectives:**

The aim of this study was to record the occupational routes of people working in an institution specialised in pain medicine/pain management as well as their motivation and job satisfaction.

**Materials and methods:**

A standardised online questionnaire was used to survey members via cooperating scientific societies and organisations.

**Results:**

Data from 398 physicians, 78 psychologists, 62 physiotherapists, three occupational therapists and 23 nursing professionals were included in the analysis. The age distribution skewed towards higher age groups, with the majority of respondents aged between 51 and 60 years. The respondents usually first came into clinical contact with pain medicine after many years of work. In the case of physicians in particular, there was a delay of almost a decade after their licence to practise. With regard to motivational factors, intrinsic aspects were rated higher than monetary ones. Work-life balance issues tended to be in the middle of the ranking. Regarding job satisfaction in pain medicine, the overall satisfaction of the respondents was high, with the lowest satisfaction ratings being given in the categories “opportunities for further career development”, “additional income options” and “recognition by superiors”. A significant proportion of respondents (1/3) stated that career changes were forthcoming—changes outside a pain medicine setting or in particular retirement.

**Conclusions:**

The results emphasise the critical perspectives regarding the future provision of care for people with chronic pain. The results provide, for the first time, an insight into the career routes of staff in the field of pain medicine and their motivations and job satisfaction, which should be considered in the discussion about ensuring personnel resources in the future.

## Hintergrund und Fragestellung

Der demografische Wandel macht auch vor der Schmerzmedizin^1^ nicht halt. Bereits seit vielen Jahren bestehen über alle beteiligten Berufsgruppen hinweg kritische Prognosen bezüglich eines drohenden Nachwuchsmangels [19]. Dies wird aufgrund bereits manifester Unterversorgung absehbar zu einem relevanten Problem in der Sicherstellung der Versorgung von Patienten mit chronischen Schmerzen führen [13, 19]. Karst et al. zeigten, dass in der ambulanten schmerzmedizinischen Versorgung bereits heute kritische Zukunftsaussichten insbesondere bzgl. Nachfolgesicherung bestehen [17]. Bei hohem Altersdurchschnitt geht derzeit ca. die Hälfte der niedergelassenen Schmerztherapeuten, die im ambulanten Bereich an der Qualitätssicherungsvereinbarung Schmerztherapie (QSV) teilnehmen und den größten Anteil an spezialisierter ambulanter schmerzmedizinischer ärztlicher Versorgung in Deutschland abdecken, nicht davon aus, einen Nachfolger für ihre Praxis zu finden [17]. Dies wird zum Teil den als unattraktiv eingeschätzten Arbeitsbedingungen zugeschrieben, insbesondere unzureichend funktionierenden Netzwerken aus Schmerz‑, Physio- und Psychotherapeuten. Nach Einschätzung der ambulant tätigen Kolleginnen und Kollegen wirkt hier jedoch auch die inhaltlich und formal fehlende Eigenständigkeit der Schmerzmedizin in Deutschland mit ein [17].

Die Betrachtung der Karrierewege, Arbeitszufriedenheit und der Einschätzung der Attraktivität von beruflichen Entwicklungsmöglichkeiten in der Schmerzmedizin wird somit zur existenziellen Frage. Die unterschiedlichen Möglichkeiten im breiten Spektrum der Fachrichtungen eröffnen vielfältige berufliche Wege in die Schmerzmedizin. Entsprechend gibt es kaum Informationen, wie Menschen in die Schmerzmedizin gelangen, mit welchen Motivationen dies erfolgt und was sie in der Schmerzmedizin hält [19]. Ziel dieser Untersuchung ist es, die Ausbildungs- und Berufswege sowie die Arbeitssituation von ärztlichen, psychologischen, physio- und ergotherapeutisch sowie pflegerisch tätigen Menschen, die in einer Einrichtung der spezialisierten schmerzmedizinischen/schmerztherapeutischen Versorgung tätig sind oder waren, zu erfassen.

## Studiendesign und Untersuchungsmethoden

In einem mehrstufigen Verfahren wurde ein Online-Fragebogen zur Erfassung der bisherigen beruflichen (Karriere‑)Wege entwickelt (siehe Supplement), mit dem Ziel, Motivationen für eine Tätigkeit in der Schmerzmedizin und die Arbeitszufriedenheit von ärztlichen, psychologischen, physio-ergotherapeutischen und pflegenden Mitarbeiterinnen und Mitarbeitern aus spezialisierten schmerzmedizinischen Einrichtungen zu erfassen. Als solche zählten:

- Schmerzambulanz/Kopfschmerzambulanz
- Schmerzklinik/Schmerztagesklinik unabhängig vom Schwerpunkt
- Schmerzmedizinische/schmerztherapeutische Schwerpunkt‑/Fachpraxis
- Rehabilitationseinrichtung mit Schmerzschwerpunkt
- Andere Kooperationsform in der spezialisierten schmerzmedizinischen/schmerztherapeutischen Versorgung

Die endgültige Fragebogenversion umfasste folgende Kapitel, die in etwa analog in allen Berufsgruppen abgefragt wurden (siehe Supplement)

- Allgemeine Qualifikation
- Arbeitserfahrung und schmerzmedizinischer Karriereweg
- Aktuelle Beschäftigungssituation und Tätigkeit
- Motivation und Arbeitszufriedenheit
- Karriereentwicklung und berufliche Veränderungen
- Demografie

Dabei wurde auch erfasst, in welchen Versorgungsformen aktuell gearbeitet wird und in welchen die schmerzmedizinische Weiterbildung erfolgte (ambulante Versorgung Hochschulambulanz [= HSA], ambulante Versorgung MVZ/Schwerpunkt‑/Fachpraxis [= amb. Praxis], ambulante Versorgung über Ermächtigung [EM], teilstationäre Assessments [= T-ASS], teilstationäre interdisziplinäre multimodale Schmerztherapie [= S-TK], stationäre interdisziplinäre multimodale Schmerztherapie [= S-IMST], innerklinische Konsildienste/Schmerzversorgung/Schmerzdienst [= Schmerzdienst], sonstige).

Die Befragung erfolgte in Kooperation mit (alphabetisch):

- Arbeitsgemeinschaft schmerztherapeutischer Einrichtungen in Bayern e. V. (ASTiB)
- Berufsverband der Ärzte und Psychologischen Psychotherapeuten in der Schmerz- und Palliativmedizin in Deutschland e. V. (BVSD)
- Deutsche Gesellschaft für Psychologische Schmerztherapie und -forschung e. V. (DGPSF)
- Deutsche Migräne- und Kopfschmerzgesellschaft e. V. (DMKG)
- Deutsche Schmerzgesellschaft e. V.
- Wissenschaftlicher Arbeitskreis Schmerzmedizin der Deutschen Gesellschaft für Anästhesiologie und Intensivmedizin e. V. (DGAI)

Ein Anschreiben inkl. Befragungslink wurde von den kooperierenden Partnern ab dem 16.11.2020 an ihre Mitglieder versandt (Ausnahme BVSD, hier erfolgte der Aufruf in einem Newsletter). Ein Reminder wurde nach 3 Wochen versendet. Die Beantwortung der Befragung war 2 Monate möglich.

## Datenschutz und Ethik

Die Beantwortung erfolgte anonym. Auf die Bundeslandzugehörigkeit wurde deshalb zur Vermeidung von Rückschlüssen verzichtet, außer für Bayern aufgrund der Unterstützung über den Verteiler eines regionalen Verbands (ASTiB). Die Kooperationspartner verwendeten die jeweiligen Mitgliedsdaten zum Versand, was die Berechnung eines Rücklaufs nicht ermöglichte. Dies zum einen, da die Verbände selbst ihre Mitglieder anschrieben, die Befragung anonym war, Mitgliedschaften und Zugehörigkeiten nicht abgefragt wurden und aufgrund einer vermutlich hohen Überschneidung von Mitgliedern, die in mehreren der kooperierenden Organisationen vertreten sind, eine Abschätzung nicht sinnvoll möglich ist. Der Studiencharakter machte eine genehmigende Beratung einer Ethikkommission nicht erforderlich (Ethik-Kommission Göttingen Nr. 111024).

## Ergebnisse

### Rücklauf und Charakteristik der Befragten

**Abb. 1:**
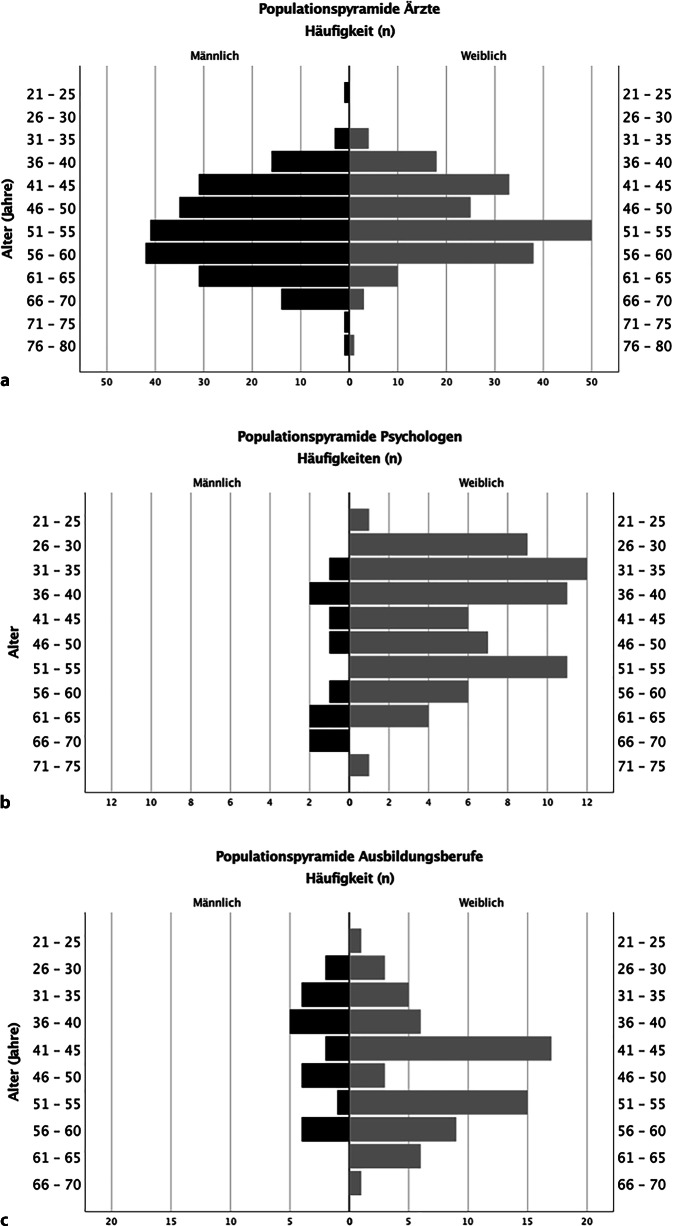
Alters- und Geschlechtsverteilung (**a** Ärzte, **b** Psychologen, **c** *P*hysiotherapeuten, *E*rgotherapeuten, *P*flegefachpersonen [PEP]). (Fehlwerte Alter und Geschlecht jeweils: „Ärzte“ *n* = 11, „Psychologen“ *n* = 3, „PEP“ Fehlwerte *n* = 6)

**Abb. 2:**
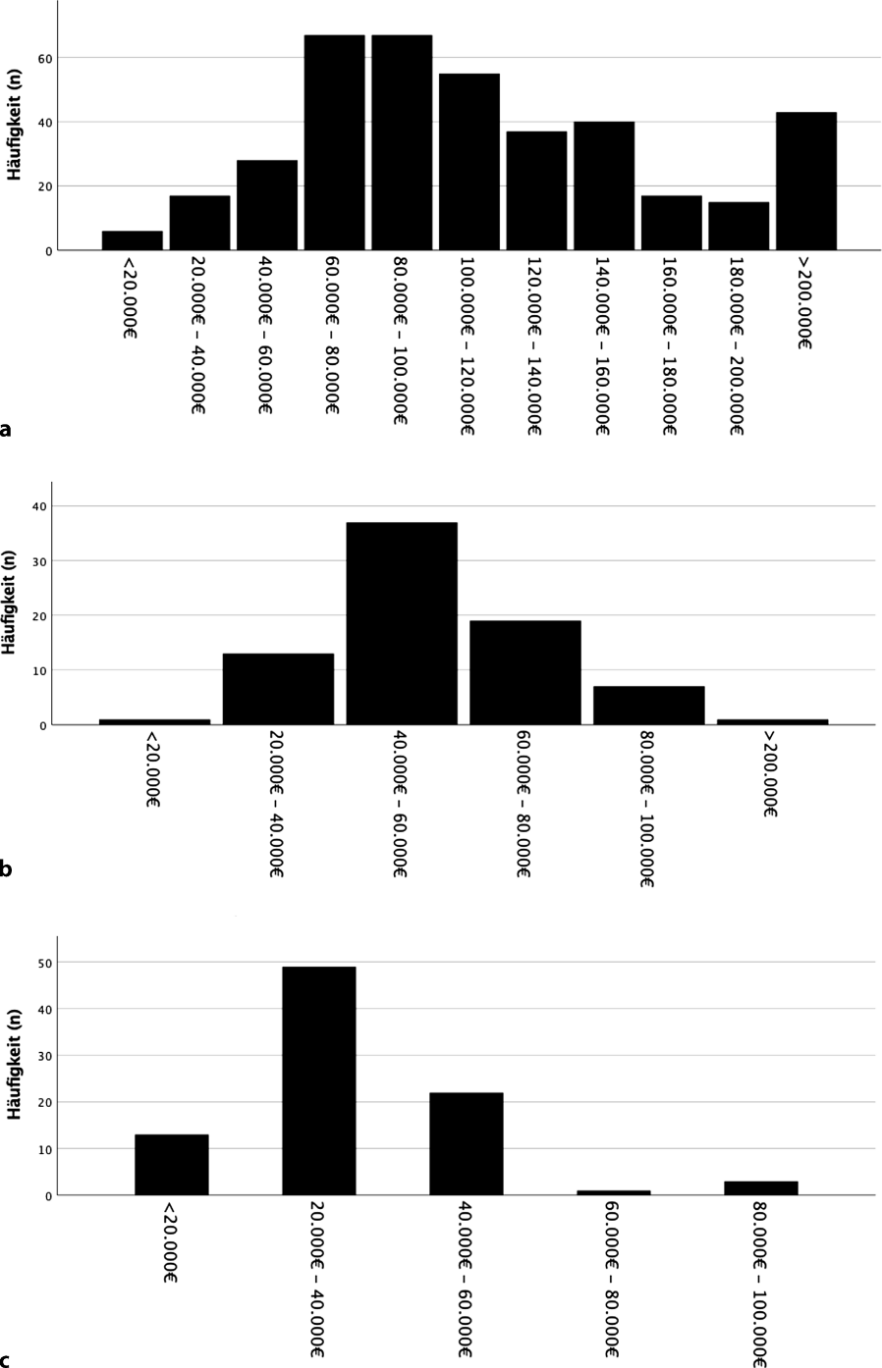
Einkommensverteilung (**a** Ärzte, **b** Psychologen, **c** *P*hysiotherapeuten, *E*rgotherapeuten, *P*flegefachpersonen [PEP])

Der Befragungslink wurde in 1276 Fällen geöffnet und von 564 Kolleginnen (*n* = 316) und Kollegen (*n* = 248)^2^ beantwortet (398 Ärzte, 78 Psychologen, 62 *P*hysiotherapeuten, 3 *E*rgotherapeuten, 23 *P*flegefachpersonen, im Folgenden als „PEP“ zusammengefasst). In der Gruppe „Ärzte“ überwogen Männer (Frauen 46 % [*n* = 178], Männer 54 % [*n* = 209], Fehlwerte *n* = 11) während in den Gruppen „Psychologen“ und „PEP“ ein deutlich höherer Anteil an Frauen vertreten war („Psychologen“: Frauen 87 % [*n* = 65], Männer 13 % [*n* = 10], Fehlwerte *n* = 3; „PEP“ Frauen 75 % [*n* = 62], Männer 25 % [*n* = 20], Fehlwerte *n* = 6). Die Altersverteilung unterschied sich ebenfalls, mit hohem Anteil in älteren Altersgruppen (51–60 Jahre) bei Ärzten (Abb. 1). Ebenso zeigten sich erwartungsgemäß unterschiedliche Einkommensverteilungen (Abb. 2).

#### Charakterisierung Ärzte

Zum Befragungszeitpunkt waren 89 % der antwortenden Ärzte in der spezialisierten Schmerzmedizin aktiv tätig, 8 % in einem anderen klinischen Kontext (1 % berentet, aber klinisch noch aktiv, 3 % nichtklinische Tätigkeit bzw. bereits im Ruhestand). Ärzte waren meist in anästhesiologischen Kliniken angestellt, zum größten Teil bei Maximalversorgern und Regelversorgern (51 % öffentliche, 29 % private und 20 % freigemeinnützige Träger; 57 % Lehrkrankenhäuser, 28 % Universitätskliniken). Die Klinikärzte gaben meist an, in einem Subbereich einer übergeordneten Abteilung unter Leitung eines Sektionsleiters bzw. bereichsleitenden Oberarztes organisiert zu sein (53 %); 38 % waren in einer eigenständigen schmerzmedizinischen Abteilung mit Chefarzt und 9 % in einem Versorgungsbereich ohne gesonderte Leitung tätig. 86 % arbeiteten an Einrichtungen, die zur Weiterbildung zur speziellen Schmerztherapie befugt waren. Selbst weiterbildungsberechtigt waren 47 % der antwortenden Klinikärzte; davon 42 % für 12 Monate. Die Mehrheit der Klinikärzte (56 %) machte keine Dienste bzw. ausschließlich Dienste in der Schmerzmedizin, 43 % erbrachten Bereitschafts‑, Ruf- oder Hintergrunddienste außerhalb der schmerzmedizinischen Versorgung (meist anästhesiologisch mit 74 %).

Unter den antwortenden niedergelassenen Ärzten (*n* = 138; 76 % Fachärzte für Anästhesiologie, 10 % Allgemeinmedizin, 6 % Neurologie) gaben 79 % an, eine Kassenzulassung zu besitzen. 14 % waren als persönlich Ermächtigte tätig und 7 % in reiner Privatpraxis. Für 98 % der hier berücksichtigten kassenärztlichen Einrichtungen wurde angegeben, dass dort zur Zusatzbezeichnung spezielle Schmerzmedizin weitergebildet würde, dies meist (85 %) für 12 Monate, 53 % waren weiterbildungsberechtigt.

#### Charakterisierung Psychologen

94 % der antwortenden Psychologen waren zum Zeitpunkt klinisch aktiv in der Schmerzmedizin tätig (2 % weiter aktiv nach Berentung bzw. 1 % in einem anderen klinischen Kontext, 5 % waren zum Befragungszeitpunkt klinisch nicht aktiv bzw. bereits im Ruhestand). Die meisten Psychologen arbeiteten in einer eigenständigen schmerzmedizinischen Klinik (mit eigenem Chefarzt, 31 %), 20 % in einer Praxis, 19 % in einer universitären und 15 % in einer nichtuniversitären anästhesiologischen Klinik, 5 % in einer nichtuniversitären psychiatrisch-/psychosomatischen Abteilung (10 % sonstiges). Von ihnen waren 13 % angestellt mit Leitungsfunktion, 68 % angestellt ohne Leitungsfunktion und 19 % selbstständig oder Partner. Ihre durchschnittliche Wochenarbeitszeit betrug 32 h und prozentual erfolgten im Mittel 67 % der Regelarbeitszeit in schmerzmedizinischer Tätigkeit.

Niedergelassene Psychologen waren zu 77 % auf einem Kassensitz tätig und zu 15 % als Privatpraxis mit Kostenerstattung ambulant organisiert. 8 % gaben einen speziellen Versorgungsauftrag an.

#### Charakterisierung Teilnehmende der Gruppe „PEP“

Teilnehmende waren zu 89 % aktiv in einer schmerzmedizinischen Einrichtung tätig (5 % in anderem Kontext, 1 % nach Berentung weiterhin aktiv und 6 % in keiner klinischen Tätigkeit bzw. im Ruhestand). 38 % waren in einer eigenständigen schmerzmedizinischen Klinik tätig, 19 % in einer universitären bzw. 10 % in einer nichtuniversitären anästhesiologischen.

### Qualifikations- und Karrierewege Ärzte

#### Qualifikation

Antwortende Ärzte waren in 99 % Fachärzte (davon mit 76 % am häufigsten für Anästhesiologie); 13 % aller Fachärzte besaßen noch einen zweiten Facharzttitel. Die Erlangung der zuerst erworbenen Facharztanerkennung lag zum Befragungszeitpunkt 16 ± 9 (min. 0, max. 46) Jahre zurück. 86 % führten die Zusatzbezeichnung (ZB) spez. Schmerztherapie, jeweils 5 % gaben an, bereits die Weiterbildungszeit absolviert zu haben oder sich noch in dieser zu befinden. Die am zweithäufigsten geführte ZB war die der Palliativmedizin (39 %, weitere: Notfallmedizin 29 %, Akupunktur 22 %, Intensivmedizin 18 %, manuelle Medizin 17 %, Suchtmedizin 12 %, Psychotherapie 9 %, Naturheilverfahren 4 %, ärztliches Qualitätsmanagement 4 %, Sportmedizin 3 %, physikalische Therapie und Balneologie 2 %, alle anderen ≤ 1 %).

**Abb. 3:**
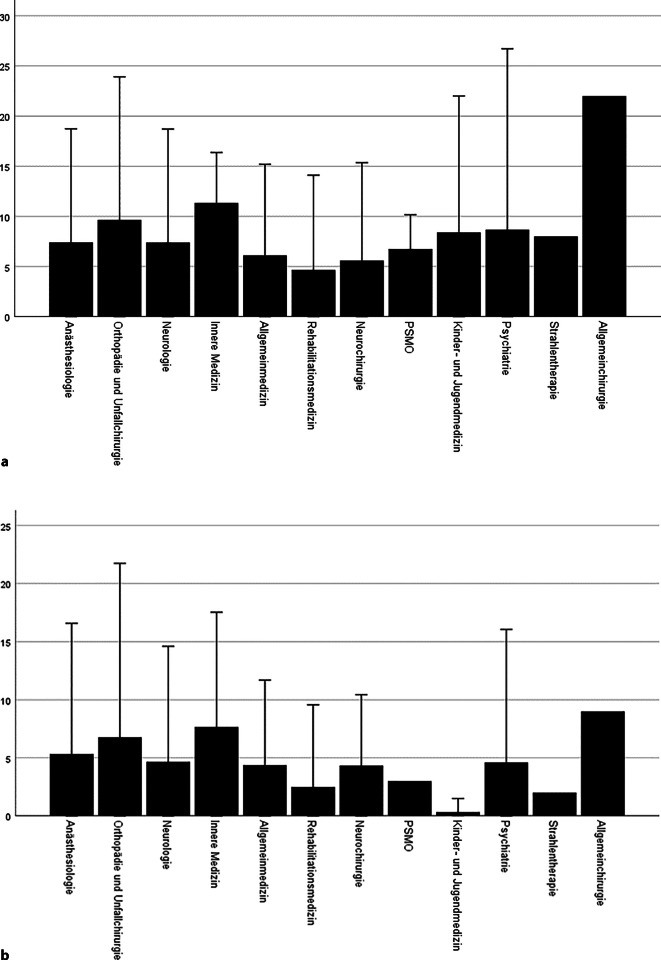
Beginn der Weiterbildung zur speziellen Schmerztherapie (**a**) nach Approbation, (**b**) nach Facharztqualifikation

Ärzte gaben an, die Weiterbildung zur ZB spez. Schmerztherapie 9 ± 6 Jahre nach ihrer Approbation begonnen zu haben (Abb. 3). Die meisten Fachärzte (71 %) hatten die Weiterbildungszeiten zur Zusatzbezeichnung erst nach Abschluss der Facharztweiterbildung absolviert, 22 % zum Teil und 7 % vollständig vor Erlangen des Facharzttitels. Die Weiterbildungszeit wurde zumeist in einer (78 %), in zwei (19 %) und nur zu einem geringen Teil in drei oder mehr Einrichtungen absolviert (3 %). Sie erfolgte zum Großteil in einer anästhesiologischen Abteilung (Mehrfachantworten; 40 % universitäre Abteilung, 33 % nichtuniversitäre Klinik/Abteilung für Anästhesiologie, 23 % eigenständige schmerzmedizinische Klinik, 14 % Schwerpunktpraxis/MVZ und Einzelpraxis, 5 % neurologische Kliniken [2 % universitär, 3 % nichtuniversitär], sonstige 9 %). Am häufigsten vertretene Versorgungsformen, in denen diese stattfand, waren S‑IMST und Schmerzdienste (Tab. 1). 33 % der Ärzte mit Zusatzbezeichnung gaben an, dass sie ihre Weiterbildung ohne Tätigkeit in teil- und/oder stationärer interdisziplinärer multimodaler Schmerztherapie absolviert hatten.

**Tab. 1:** Versorgungsformen Weiterbildungszeit, Arbeitserfahrung, aktuelle Tätigkeit

	Versorgungsformen, in denen unter anderen die Weiterbildungszeit stattfand			Versorgungsformen, in denen im Laufe der schmerzmedizinischen Tätigkeit Arbeitserfahrung gesammelt wurde			Versorgungsformen der aktuellen Einrichtung		
	A	P	PEP	A	P	PEP	A	P	PEP
Innerklinische Konsildienste/Schmerzdienst	50 (192)	9 (4)	–	63 (250)	–	11 (10)	53 (187)	21 (16)	19 (15)
Stationäre interdisziplinäre multimodale Schmerztherapie	54 (209)	52 (24)	–	68 (269)	61 (48)	64 (56)	52 (185)	49 (37)	62 (48)
Ambulante Versorgung MVZ/Schwerpunkt‑/Fachpraxis	32 (124)	26 (12)	–	43 (169)	22 (17)	13 (11)	41 (144)	40 (30)	17 (13)
Teilstationäre interdisziplinäre multimodale Schmerztherapie	28 (108)	28 (13)	–	38 (150)	46 (36)	38 (33)	32 (112)	45 (34)	45 (35)
Stationäre Assessments	–	–	–	–	–	–	31 (111)	19 (14)	27 (21)
Teilstationäre Assessments	19 (71)	17 (8)	–	28 (112)	32 (25)	22 (19)	28 (99)	39 (29)	39 (30)
Ambulante Versorgung über Ermächtigung	–	–	–	28 (111)	6 (5)	11 (10)	25 (87)	4 (3)	9 (7)
Ambulante Versorgung Hochschulambulanz	40 (154)	24 (11)	–	42 (166)	20 (16)	10 (9)	18 (64)	9 (7)	17 (13)
Stationäre Rehabilitation	6 (21)	24 (11)	–	9 (36)	18 (14)	9 (8)	3 (11)	5 (4)	6 (5)
Ambulante Rehabilitation	2 (7)	7 (3)	–	3 (10)	5 (4)	3 (3)	1 (5)	3 (2)	4 (3)

% (*n*)

*A* Ärzte, *P* Psychologen, *PEP* Physiotherapeuten, Ergotherapeuten, Pflegefachpersonen

#### Arbeitserfahrung in der Schmerzmedizin

Ärzte waren nach Approbation nach 8 ± 6 Jahren (max. 30) erstmals in einer spezialisierten schmerzmedizinischen Einrichtung klinisch aktiv tätig bzw. haben zuvor 5 ± 6 (min. 0; max. 28) Jahre bereits als Facharzt gearbeitet (Abb. 3). Nur 32 % der Ärzte gaben an, bereits vor der Facharztprüfung in einer spezialisierten schmerzmedizinischen Einrichtung tätig gewesen zu sein.

Die in der Schmerzmedizin zum Befragungszeitpunkt tätigen Ärzte gaben an, 11 ± 9 (min. 0; max. 40) Jahre ohne relevante Unterbrechungen in der Schmerzmedizin tätig gewesen zu sein und kumuliert 13 ± 9 (min. 0; max. 40) Jahre klinische schmerzmedizinische Erfahrung zu haben. Klinische Erfahrung bestand am häufigsten aus der Tätigkeit in der S‑IMST, dem Schmerzdienst, der Tätigkeit in einer amb. Praxis oder Hochschulambulanz (HSA, Tab. 1). Immerhin hatten 19 % aller antwortenden Ärzte in ihrem beruflichen Werdegang keine aktive klinische Erfahrung in teilstationärer und/oder stationärer interdisziplinärer multimodaler Schmerztherapie (17 % derjenigen mit ZB).

Der überwiegende Teil der Ärzte (43 %) war innerhalb der schmerzmedizinischen Beschäftigung beim selben Arbeitgeber tätig. Ein Arbeitgeberwechsel im stattgehabten Karriereweg fand meist nur einmal (31 %) statt (zweimal 13 %, dreimal und mehr 13 %). Der letzte Wechsel lag meist (56 %) bereits mindestens 5 Jahre zurück (kategoriale Abfrage; max. 6 Monate 6 %, 7–12 Monate 8 %, 13–24 Monate 8 %, 25–60 Monate 23 %).

### Qualifikations- und Karrierewege Psychologen

#### Qualifikation

Von den teilnehmenden Psychologen (71 % [*n* = 56] Diplom, 29 % [*n* = 23] Master) waren 77 % (*n* = 61) approbiert. Die Approbation lag 11 ± 8 (min. 0; max. 21) Jahre vom Befragungszeitpunkt zurück. 88 % (*n* = 54) der Approbierten hatten eine Fachkunde in Verhaltenstherapie, 8 % (*n* = 5) in tiefenpsychologisch fundierter Psychotherapie; 4 % hatten keine Fachkunde.

Die Qualifikation spezielle Schmerzpsychotherapie hatten 56 % (*n* = 34) der antwortenden Psychologen und 21 % (*n* = 13) waren zum Befragungszeitpunkt noch in der Weiterbildung dazu. Die Zusatzqualifikation wurde 9 ± 7 (max. 23) Jahre nach dem Studienabschluss erlangt. In 74 % (*n* = 35) wurde die Weiterbildungszeit nach der Approbation absolviert, in 15 % (*n* = 7) zum Teil und in 11 % (*n* = 5) vollständig vor der Approbation. Die Weiterbildung erfolgte mehrheitlich (72 %) an einer einzigen, selten an zwei (17 %) bzw. an drei oder mehr (11 %) Einrichtungen (meistens eine eigenständige schmerzmedizinische Klinik oder eine Rehabilitationseinrichtung). Die häufigste Versorgungsform dabei war die S‑IMST (Tab. 1).

#### Arbeitserfahrung in der Schmerzmedizin

Psychologen gaben an, 5 ± 5 (min. 0; max. 22) Jahre nach ihrem Studienabschluss bzw. 4 ± 5 (min. 0; max. 16) Jahre nach Approbation das erste Mal in einer spezialisierten schmerztherapeutischen Einrichtung klinisch tätig gewesen zu sein; 53 % der approbierten Psychologen gaben dies bereits für die Zeit vor der Approbation an. Die in der Schmerzmedizin zum Befragungszeitpunkt tätigen Psychologen gaben an, bereits 9 ± 8 (min. 0; max. 34) Jahre ohne relevante Unterbrechungen in der Schmerzmedizin tätig gewesen zu sein und kumuliert 10 ± 9 (min. 0, max. 34) Jahre klinische schmerzmedizinische Erfahrung zu haben, welche mehrheitlich aus der Tätigkeit in einer (60 %), zwei (22 %) bzw. drei und mehr (18 %) Einrichtungen resultierte. Auch hier war die S‑IMST die häufigste Versorgungsform (Tab. 1).

Deutlich häufiger als bei den Ärzten war der überwiegende Teil der Psychologen (63 % versus 43 %) bei einem Arbeitgeber schmerzmedizinisch tätig gewesen; Arbeitgeberwechsel fanden größtenteils (20 %) nur einmalig statt (zweimalig 13 %, dreimalig und öfter 4 %). Der letzte Wechsel lag zu 48 % länger als 5 Jahre zurück (max. 6 Monate keiner, 7–12 Monate 3 %, 13–24 Monate 7 %, 25–60 Monate 41 %).

### Qualifikations- und Karrierewege Physio- und Ergotherapeuten/Pflegefachpersonen

#### Qualifikation

Antwortende Physiotherapeuten (*n* = 62) beendeten ihre Ausbildung vor 20 ± 11 (min. 1; max. 44) Jahren. 66 % hatten die Weiterbildung spezielle Schmerzphysiotherapie angeschlossen. Diese wurde im Mittel 17 ± 10 (min. 0; max. 39) Jahre nach dem Berufsabschluss abgeschlossen. 27 % hatten einen akademischen Abschluss. Ergotherapeuten hatten ihre Ausbildung vor 5 ± 2 (min. 3; max. 6) Jahren abgeschlossen. Pflegefachpersonen hatten vor 24 ± 8 (min. 1; max. 36) Jahren ihre Ausbildung abgeschlossen und hatten in 77 % eine abgeschlossene Fachweiterbildung (Anästhesie/Intensivpflege 50 %, Palliativ- und Hospizpflege 31 %, Onkologie 13 %, andere 6 %). 11 % verfügten zudem über einen akademischen Abschluss. 91 % hatten die Qualifikation zum pflegerischen Schmerzexperten (bis zum Jahr 2020, ohne Differenzierung).

#### Arbeitserfahrung in der Schmerzmedizin

Mitarbeitende dieser Gruppe waren 11 ± 9 (min. 0; max. 37) Jahre nach Ausbildungsabschluss das erste Mal in einer spezialisierten schmerzmedizinischen Einrichtung klinisch aktiv, und zuletzt ohne relevante Unterbrechungen 9 ± 7 (min. 0; max. 36) Jahre klinisch dort tätig. Im Mittel bestanden 9 ± 7 (min. 0; max. 36) Jahre kumulierte klinische Berufserfahrung in der Schmerzmedizin, welche mehrheitlich (85 %) an einer einzigen Einrichtung gesammelt wurde (Tab. 1). Entsprechend selten gab es in dieser Berufsgruppe Arbeitgeberwechsel (17 %), die in den meisten Fällen mindestens über 5 Jahre zurücklagen.

### Motivationen für die Tätigkeit in der Schmerzmedizin

**Abb. 4:**
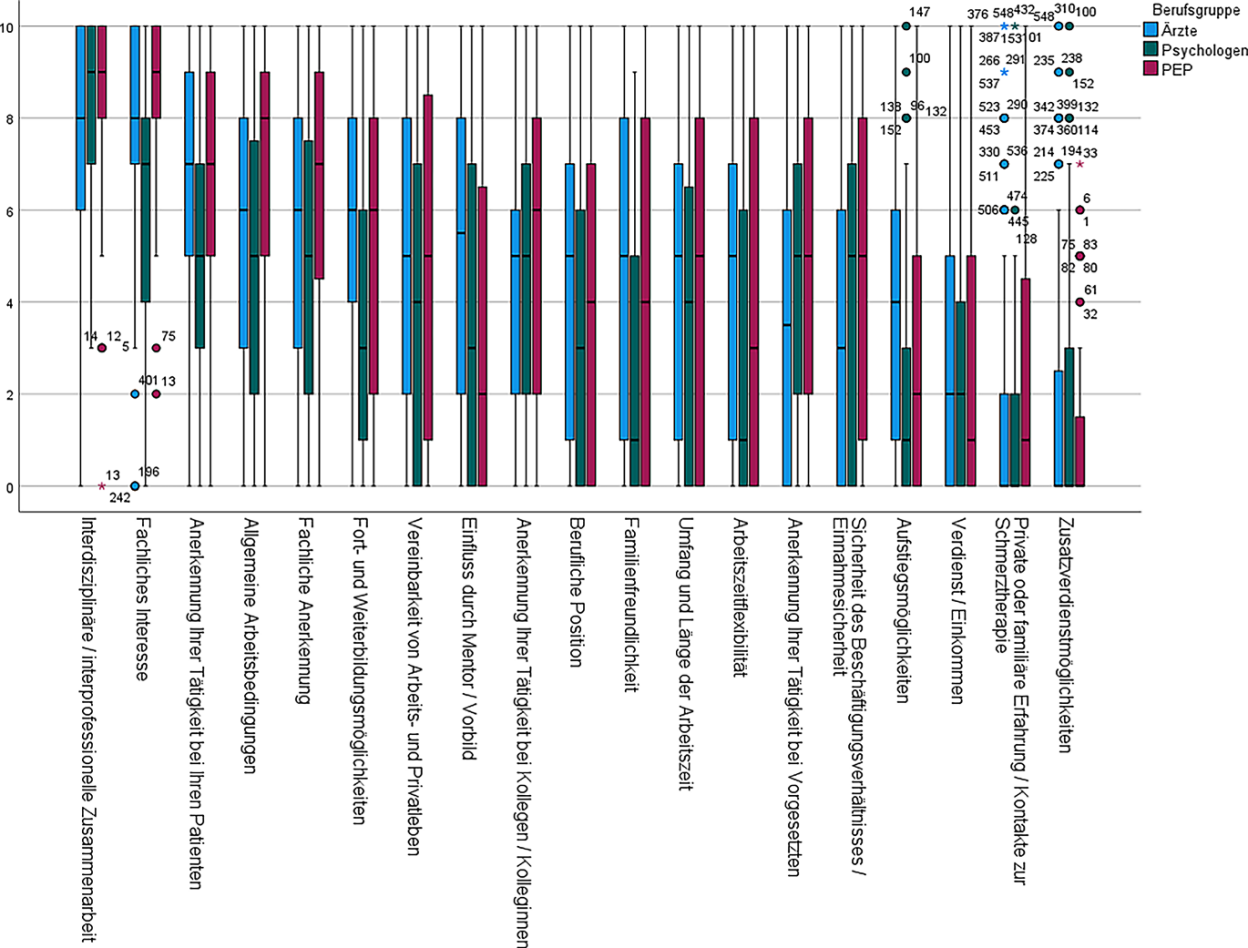
Motivationale Einflussfaktoren zur Tätigkeit in der Schmerzmedizin

Die motivationalen Einflussfaktoren zur Tätigkeit in der Schmerzmedizin wurden einzeln sehr unterschiedlich priorisiert (Abb. 4). Die Gesamtgewichtung war dabei zwischen den Berufsgruppen recht vergleichbar, wenn es auch punktuell deutliche Unterschiede zwischen einzelnen Items gab. Intrinsische Faktoren wurden am stärksten gewichtet. Faktoren wie Arbeitszeit bzw. Vereinbarkeit zwischen Arbeit und Familie/Beruf lagen eher im mittleren und monetäre Faktoren im hinteren Drittel. Von Ärzten wurde die Erlangung der ZB spezielle Schmerztherapie bzw. die damit verbundenen klinischen Erfahrungen als großer Einflussfaktor für die Tätigkeit in der Schmerzmedizin bewertet, Kontakte aus Zeiten des Studiums bzw. der Promotion am geringsten. Psychologen bewerteten „Aufstiegsmöglichkeiten“, „Verdienst/Einkommen“, „Zusatzverdienstmöglichkeiten“, „Arbeitszeitflexibilität“ und „Familienfreundlichkeit“ als geringsten Motivationsfaktor für die Tätigkeit in der Schmerzmedizin. Fachliches Interesse und Interdisziplinarität/Interprofessionalität wurden von Kolleginnen und Kollegen der „PEP“ mit am stärksten bewertet, auch hier wurden „Aufstiegsmöglichkeiten“ sowie „Verdienst/Einkommen“ gering bewertet.

### Arbeitszufriedenheit

**Abb. 5:**
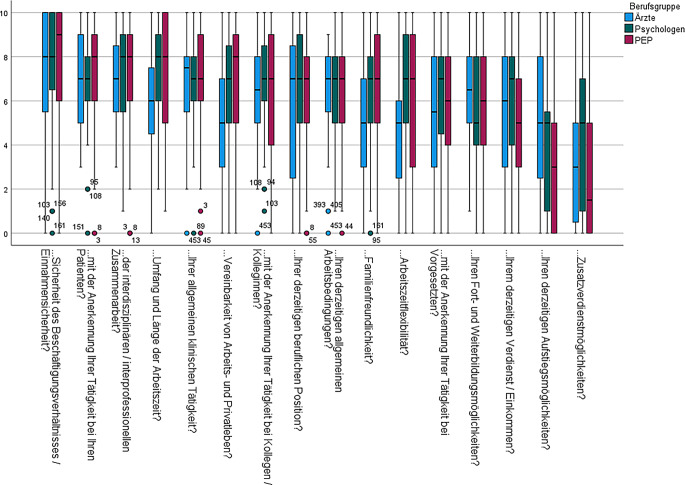
Arbeitszufriedenheit in der Schmerzmedizin

Bei der Betrachtung der Arbeitszufriedenheit zeigte sich ebenfalls, dass Aspekte zwischen Berufsgruppen tendenziell ähnlich bewertet wurden (Abb. 5). Der von allen am höchsten bewertete Aspekt war die Sicherheit des Beschäftigungsverhältnisses und des Einkommens sowie die „Interdisziplinarität und Interprofessionalität“.

Die Zufriedenheit war bei Ärzten am höchsten bzgl. „beruflicher Position“, der „klinischen Tätigkeit als Schmerzmediziner (Schmerztherapeut) im Allgemeinen“, „der allgemeinen klinischen Tätigkeit“ und der „Anerkennung der Tätigkeit bei Patienten“ gewertet. Die geringste Zufriedenheit bestand bei Ärzten bzgl. Aufstiegsmöglichkeiten, Zusatzverdienstmöglichkeiten und der Anerkennung beim Vorgesetzten. Psychologen bewerteten des Weiteren ihre Zufriedenheit bzgl. „Umfang und Länge der Arbeitszeit“ und „Arbeitszeitflexibilität“ am höchsten. „Aufstiegsmöglichkeiten“, „Fort- und Weiterbildungsmöglichkeiten“ sowie „Zusatzverdienstmöglichkeiten“ am niedrigsten. Von Antwortenden der Gruppe „PEP“ wurde neben den Flexibilitäts- und Vereinbarkeitsaspekten zudem die Zufriedenheit mit „Umfang und Länge der Arbeitszeit“ und der „Anerkennung der Tätigkeit bei Patienten“ am höchsten bewertet. Analog zu den anderen Berufsgruppen wurden auch hier „Aufstiegsmöglichkeiten“ und „Zusatzverdienstmöglichkeiten“ mit den niedrigsten Zufriedenheitswerten abgebildet.

### Karriereentwicklung und berufliche Veränderungen

Der Blick auf die Karriereentwicklung stellte sich ambivalent dar. Einerseits war die Zustimmung, Karriereziele erreicht zu haben, über alle Berufsgruppen relativ hoch. Andererseits wurden Aufstiegsmöglichkeiten in der Schmerzmedizin an der derzeitigen Arbeitsstelle als sehr niedrig bewertet. Besser war die Bewertung bezogen auf den allgemeinen Arbeitsmarkt bzw. die zukünftige finanzielle Sicherheit durch eine Tätigkeit in der Schmerzmedizin. Mit Blick auf konkrete geplante Veränderungen gab es einen kritischen Anteil Ärzte, der in naher Zukunft eine berufliche Veränderung plante (Tab. 2). Zudem stand ca. ein Viertel vor dem Ruhestand bzw. der Berentung. 34 % der befragten Psychologen und 33 % der PEP planten in naher Zukunft eine berufliche Veränderung (Tab. 2). Gründe für die Beendigung der Tätigkeit in der Schmerzmedizin waren (Freitext) unzureichende berufliche Aufstiegs- bzw. Entwicklungsmöglichkeiten (z. B. „mußte mich entscheiden, entweder weiter alleine Schmerztherapie oder im Team Leiter der Intensivstation“, „begrenzte Karrieremöglichkeiten“, „fehlende schmerzmedizinische Stellen nach Weiterbildungsrotation“, „Schließung der Einrichtung“ bzw. „Abbau von stationären Schmerzbetten durch den Klinikträger“, „keine längerfristige Perspektive“, „fehlender freier Kassensitz“, „Hausärztliche Tätigkeit und gleichzeitige Tätigkeit gemäß Schmerztherapievereinbarung nicht möglich“, „in der hiesigen Region keine Spezi. Angebote“), Wertschätzung und monetäre Aspekte (z. B. „zu wenig/extrem schlechter Verdienst“, „hohe Qualitätsansprüche und wenig Vergütung“, „Geringer Stellenwert der Schmerzmedizin […]“, „andere Fachärzte werden deutlich besser wertgeschätzt“) und gesundheitspolitische Aspekte (z. B. „KV verhindert die Zulassung als Spezialambulanz“, „Begutachtungskriterien des MDK“, „Auflagen für Zulassung zu hoch“, „Kein Facharzt für Schmerzmedizin, damit unzureichende Möglichkeit der Niederlassung“, „Als Neurologin mit Zusatz Spezielle Schmerzmedizin ist ein angestelltes ambulantes Arbeiten nach QSV sehr schwer umsetzbar, […] und ist-Hürden extrem, politisch nicht gewollt“).

**Tab. 2:** Berufliche Veränderung

	A	P	PEP
*Berufliche Veränderung konkret geplant?*			
Nein, nicht geplant	60 (232)	66 (50)	67 (55)
Ja, berufliche Veränderung konkret geplant	40 (155)	34 (26)	33 (27)
*Art der konkreten Veränderung*			
Berentung/Ruhestand	24 (37)	15 (37)	26 (7)
Wechsel in die Niederlassung/Selbstständigkeit	16 (25)	38 (25)	11 (3)
Aufstieg innerhalb der jetzigen Klinik in der Schmerzmedizin	14 (21)	12 (3)	15 (4)
Aufstieg innerhalb der jetzigen Klinik unabhängig von der Schmerzmedizin	5 (7)	12 (3)	7 (2)
Wechsel in andere schmerzmedizinische Einrichtung ohne Veränderung der Position	5 (8)	8 (2)	4 (1)
Wechsel in andere Einrichtung ohne schmerzmedizinischen Bezug ohne Veränderung der Position	3 (3)	–	7 (2)
Wechsel in andere schmerzmedizinische Einrichtung mit Verbesserung der Position in der Schmerzmedizin	10 (16)	–	4 (1)
Wechsel in andere Einrichtung mit Verbesserung der Position unabhängig von der Schmerzmedizin	8 (13)	–	26 (7)
Erweiterung/Expansion der Niederlassung/Selbstständigkeit	14 (21)	15 (4)	–
Wechsel in Forschung	1 (2)	–	–

% (*n*)

*A* Ärzte, *P* Psychologen, *PEP* Physiotherapeuten, Ergotherapeuten, Pflegefachpersonen

## Diskussion

Die Ergebnisse liefern erstmals einen Einblick in die Wege von Mitarbeitenden in die Schmerzmedizin, deren Motivationen und Arbeitsplatzzufriedenheit. Die Befragten kamen meist erst nach Jahren der Berufstätigkeit klinisch mit der Schmerzmedizin in Kontakt. Hinsichtlich motivationaler Faktoren werden eher intrinsische Aspekte gewichtet als monetäre. Bzgl. der Arbeitszufriedenheit in der Schmerzmedizin zeichnet sich berufsgruppenübergreifend ein Bild mit insgesamt höheren Werten an Zufriedenheit auf den genutzten Skalen ab. Reduzierte berufliche Weiterentwicklungs- und Aufstiegsmöglichkeiten sind jedoch relevant. Über 1/3 der Befragten gab an, dass berufliche Änderungen anstehen, teils der Rentenbeginn, teils andere Versorgungsbereiche, was die Wahrnehmung kritischer Zukunftsaussichten hinsichtlich zukünftiger Versorgungssicherung von Menschen mit chronischen Schmerzen unterstreicht.

### Limitationen

Wichtigste Limitation und typische Einschränkung für Umfragedaten sind mögliche Verzerrungen durch Selektionsbias. Der exakte Rücklauf unserer Befragung ist nicht darstellbar, da die kooperierenden Verbände jeweils selbst ihre Mitglieder anschrieben und zudem von höherer Überschneidung auszugehen ist. Immerhin wurde in fast 50 % der Fälle des Öffnens des Links der Fragebogen beantwortet. Antwortende waren meist Ärzte und nur in kleinerem Umfang Berufsgruppen, die in Deutschland zumindest historisch einen Berufsausbildungsabschluss machten. Das kann daran liegen, dass diese ggf. in der Breite nicht in den teilnehmenden Gesellschaften vertreten sind oder sich nicht vom Titel angesprochen fühlten.

Für die deutlichen Unterschiede in der Altersverteilung zwischen den Berufsgruppen bleibt unklar, inwieweit dies repräsentativ oder ein Selektionsbias ist (z. B. bei hohem Anteil leitender oder niedergelassener Ärzte). Der gerade unter Ärzten hohe Anteil höherer Altersgruppen könnte auch die berufspolitisch beschriebene „Überalterung“ widerspiegeln [19] und zeigt sich bei den Antwortenden der anderen Berufsgruppen weniger. Die Verteilung der Antwortenden auf Einrichtungen in Bezug auf die Trägerschaft überrepräsentierte im Vergleich des Bundesdurchschnitts leicht öffentliche Träger, freigemeinnützige waren dagegen etwas unterrepräsentiert [12]. Die Verteilung der Facharztzugehörigkeit entspricht zumindest in etwa der Verteilung unter den Mitgliedern der Deutschen Schmerzgesellschaft. Ebenso zeigte sich, dass die Altersverteilung der Antwortenden der Altersverteilung der Mitglieder der Deutschen Schmerzgesellschaft entsprach. Der Zeitpunkt der Befragung Ende des ersten Jahres der Coronapandemie vermag ggf. die Angaben zu Motivationen und Arbeitszufriedenheit beeinflusst haben – unklar, in welche Richtung. Um diesen Bias gering zu halten, hatten wir bewusst die Fragen teils allgemeiner gehalten und stets auf die Tätigkeit in der Schmerzmedizin bezogen.

### Später Kontakt als kritischer Faktor in der ärztlichen Nachwuchs- und Versorgungskrise

Schmerzmedizin ist ein interdisziplinärer Versorgungsbereich ohne eigene Facharztbezeichnung und Berücksichtigung bei der Versorgungsplanung (z. B. Krankenhausbedarfsplan; [21]). Nur Fachärzte können die entsprechende Zusatzbezeichnung erlangen. Ein Beginn der Weiterbildung vor der Facharztprüfung verlängert die erforderlichen Ausbildungszeiten [9]. Unsere Ergebnisse machen deutlich, dass ein persönlicher Kontakt zur Schmerzmedizin oder Beginn der Weiterbildung häufig später im Laufe der Berufstätigkeit stattfindet und somit bei vielen (gerade ärztlichen) Kolleginnen und Kollegen in die Mitte bzw. das Ende der Dreißiger-Lebensjahre fällt; eine Zeit, die meist stark von Familienaufbau, Kinderbetreuung etc. geprägt ist und damit von Limitationen in der Flexibilität eines neuen Karrierewegs, insbesondere in Bezug auf (Orts‑)Wechsel und persönlichen Neuanfang. Zudem erfolgte ggf. bereits eine fachliche Schwerpunktsetzung, die sich unmittelbar aus der Facharztweiterbildung ergab bzw. leichter zugänglich war. Andererseits vermag gerade die meist elektive Schmerzmedizin ein relativ hohes Maß flexibler und familienfreundlicher Arbeitszeitmodelle zu bieten. Eine Umfrage von anästhesiologischen Ärzten in Weiterbildung zeigte, dass im Vergleich zu anderen Zusatzqualifikationen anteilig das niedrigste Interesse für die ZB spezielle Schmerztherapie bestand [4]. Jedoch war der Erwerb der ZB den Angaben zufolge der „Wendepunkt“ in Richtung Schmerzmedizin und weniger die Vorerfahrung aus Studium und fachärztlicher Weiterbildung [20].

### Weiterbildung

Deutlich wird, dass gerade die Universitätskliniken bzw. fachbezogen die anästhesiologischen Abteilungen den Großteil an ärztlicher (Zusatz‑)Weiterbildung innerhalb des erfassten Kollektivs abgedeckt haben. Obwohl fast die Hälfte der Niedergelassenen angab, weiterbildungsberechtigt zu sein, spiegelt sich das nicht in den Angaben wider, wo Weiterbildung absolviert wurde. Hierin zeigen sich vermutlich die oft geäußerten Nöte, dass zum einen Weiterbildung in der Praxis ein relevanter Kostenfaktor ist und Weiterbildende das wirtschaftliche Risiko persönlich tragen. Im Kontext der Allgemeinmedizin und teils auch der anderer Facharztrichtungen bestehen Förderungen von Weiterbildungsstellen, z. B. durch die Kassenärztlichen Vereinigungen (KV), um den Versorgungsdefiziten entgegenzuwirken [2, 5]. Über diese wird ein Teil der Ausbildungskosten in der ambulanten Weiterbildung von Praxen gedeckt [14]. Förderfähigkeit besteht oft jedoch nur für Facharztgruppen und ist somit für die Schmerzmedizin nicht wirksam. Handlungsdruck besteht, da derzeit anzunehmen ist, dass für die Versorgungssicherung ein potenziell verheerendes Zusammenwirken durch Verluste stationärer Einrichtungen (z. B. durch unklare Vorgaben bei der Krankenhausreform und anvisierter Ambulantisierung sowie Klinikschließungen wegen Überschuldung und Insolvenzen) ein Entfallen von Weiterbildungsstätten bedingt, bei gleichzeitig hohem Altersdurchschnitt und bereits manifesten Nachfolgeproblemen [1, 6–8, 17]. Ein pragmatischer Ansatz wäre, auch in Kliniken Weiterbildungsstellen per Förderung, z. B. durch die KV oder Krankenkassen, zu sichern und/oder zusätzlich einzurichten. Frühere und konsequente Integration der Schmerzmedizin in die Weiterbildung könnte ebenfalls hilfreich sein, mehr Ärztinnen und Ärzte zu qualifizieren und für die Schmerzmedizin zu gewinnen.

Bei den Psychologen war die Zahl der weitergebildeten bzw. sich in Weiterbildung befindlichen Kolleginnen und Kollegen recht hoch. Die spezielle Schmerzpsychotherapie wurde im Vergleich zur ärztlichen Zusatzqualifikation meist etwas früher im Karriereweg erlangt. Für Psychologen ist nach der Reform der Psychotherapeutenausbildung eine weitreichende Änderung eingetreten; es gibt jetzt den Studiengang „Psychotherapie“, der mit Master-Abschluss und Approbation endet. Daran schließt sich eine 5‑jährige Weiterbildungszeit an, die mit der Qualifikation „Fachpsychotherapeut“ (vergleichbar zur Facharztqualifikation) beendet wird [18]. Schon während der Weiterbildungszeit kann zukünftig mit einer Bereichsvertiefung „spezielle Schmerzpsychotherapie“ begonnen werden. Der Anteil an schmerzspezifisch Qualifizierten in der Gruppe der Physio- und Ergotherapeuten sowie Pflegefachpersonen war hoch. Im Gegensatz zur ärztlichen und psychologischen Qualifikation braucht es hierzu keine spezifische Weiterbildungsstätte.

### Arbeitserfahrung

Konkrete klinische Arbeitserfahrungen wurden in fast allen Berufsgruppen über mehrere Versorgungsformen gemacht. Nicht spezifisch für diese Befragung, aber ein systemimmanentes Problem ist, dass Berufserfahrung in der ärztlichen Weiterbildung nicht zwingend Fähigkeiten der IMST vermittelt. Das führt qualifikatorisch ad absurdum, wenn man bedenkt, dass z. B. ärztlich im Anschluss an die Weiterbildung sofort eine IMST-Einrichtung geleitet werden kann. Unsere Ergebnisse spiegeln wider, dass Weiterbildung innerhalb der IMST, gewissermaßen des „Goldstandards“ der Schmerztherapie, in den beruflichen Qualifikations- und Erfahrungswegen der Betroffenen oft zu kurz kommt. Dies sollte ggf. in zukünftigen Überlegungen der Musterweiterbildungsordnungen Berücksichtigung finden.

### Motivationale Faktoren und Arbeitszufriedenheit

Die Gesamtgewichtung der einzelnen Faktoren hinsichtlich Motivation für die Arbeit in der Schmerzmedizin und der Zufriedenheit war zwischen den Berufsgruppen in der Gesamtheit recht vergleichbar, wenn es auch punktuell deutliche Unterschiede zwischen den Berufsgruppen in einzelnen Items gab. Ein hervorzuhebender und für die Schmerzmedizin als Arbeitsbereich sprechender Punkt ist die sich in unseren Ergebnissen abzeichnende hohe Zufriedenheit der Befragten mit der Arbeit an sich. Diese ist in der klinischen Versorgung heutzutage nicht selbstverständlich [15]. Insgesamt wurden am stärksten eher intrinsische Faktoren stark gewichtet. Dabei kam sowohl bzgl. Motivationen als auch bei der Bewertung der Arbeitszufriedenheit die Arbeit im interdisziplinären und interprofessionellen Team zum Tragen – das Kernprinzip jeder IMST [3]. Andererseits sollten die zum Teil ambivalenten Ergebnisse der Bewertung der Karriereentwicklung kritisch wahrgenommen werden. Ein relevanter Anteil der Ärzte und Psychologen gab an, dass eine berufliche Veränderung ansteht. Dies sind Hinweise für die möglicherweise auch berufsgruppenspezifisch limitierte Attraktivität einer spezialisierten Tätigkeit in der schmerzmedizinischen Versorgung. Der z. B. „klassische“ Perspektive vom Assistenten zum Facharzt, zum Oberarzt, zum Chefarzt ist hier aufgrund des Umstands, dass die Schmerzmedizin meist innerhalb einer übergeordneten Abteilung eingebunden ist, schwierig. Ebenso kritisch wie vielsagend ist in diesem Rahmen die im Verhältnis niedrigste Bewertung der erfahrenen Anerkennung von Vorgesetzten.

Für die nichtärztlichen Kolleginnen und Kollegen scheint die Frage nach beruflichen Aufstiegsmöglichkeiten noch schwieriger, diese werden in den Angaben dieser Kolleginnen und Kollegen gar nicht erst gesehen. Damit einhergehend wurden auch „Verdienst/Einkommen“ und „Zusatzverdienstmöglichkeiten“ als Motivatoren mit am geringsten bewertet. Hier wird die wünschenswerte und zunehmende Akademisierung bisher in Deutschland „klassischer“ Ausbildungsberufe perspektivisch vermutlich eine besondere Herausforderung sein. Sicherlich braucht es für die Zukunft neben sich verändernden Aufgabenprofilen unter Berücksichtigung der neuen Kompetenzen auch eine entsprechende finanzielle Berücksichtigung, um die gesundheitspolitisch gewollte Akademisierung zu sichern und zu verstetigen [22]. Überraschenderweise spielten Flexibilität und familienfeindliche Aspekte insgesamt über alle Berufsgruppen nur eine mittlere Rolle in der Bewertung der Tätigkeit.

### Gesundheitspolitische Implikationen

Auch wenn eher intrinsische Motivationsfaktoren überwogen, unterstützen die Ergebnisse dieser Befragung die Hypothese, dass es neben früherer klinischer Einbeziehung auch attraktiver Karrierewege bedarf, um entsprechend auf dem engen Markt an Fachkräften und hochqualifizierten Menschen für die schmerzmedizinische Versorgung Personal gewinnen zu können. Das gilt innerklinisch, wo oft eine von wenigen verfügbaren Oberarztstellen das Höchste des Erreichbaren ist, als auch für wissenschaftliche Stellen und Professuren. Ökonomische Zwänge bei Unterfinanzierung von Schmerzambulanzen und z. T. Schmerzkliniken ist somit nicht nur kurzfristig eine Gefahr der Versorgung, sondern wirkt auch langfristig.

Schaut man sich die derzeitig im Fokus stehenden gesundheitspolitischen Themen an, bestehen Zweifel, dass die Versorgung von Menschen mit chronischen Schmerzen eine relevante Priorität hat [7, 8]. Im Gegenteil! Durch die gesundheitspolitisch zu verantwortende unklare zukünftige sektorale Einordnung und Finanzierung der IMST besteht eher Unklarheit zu wirtschaftlichen Perspektiven, was vermutlich negative Auswirkungen auf die Attraktivität der Schmerzmedizin als berufliches Wirkungs- und Entwicklungsfeld haben kann. Es ist zu erwarten, dass bei der sich derzeit bereits darstellenden Verschärfung der Unterversorgung am Ende ggf. eher (zum Teil) kostenintensivere Gesundheitsressourcen, die Chronifizierung im Zweifel nicht verbessern, sondern ggf. eher verstärken, zunehmend von Menschen mit chronischen Schmerzen genutzt werden [10, 11]. Es ist die Verantwortung der Gesundheitspolitik, die große Zahl Betroffener nicht „im Regen stehen zu lassen“, sondern die Rahmenbedingungen für eine qualitativ hochwertige und zeitgemäße, dem Krankheitsbild gerecht werdende abgestufte Versorgung sicherzustellen [16]. Doch dazu braucht es qualifiziertes Personal mit entsprechend attraktiven Karrierewegen.

## Schlussfolgerung

Trotz hoher Arbeitszufriedenheit und intrinsischer Motivation bei Mitarbeitenden von speziellen schmerzmedizinischen Einrichtungen sind fehlende Aufstiegsmöglichkeiten und ein hoher Anteil von Mitarbeitenden mit anstehenden beruflichen Veränderungen oder Berentung ein kritisches Thema für die zukünftige Sicherstellung der Patientenversorgung. Verschärft wird dies durch die der Gesundheitspolitik anzulastende Verunsicherung durch unklare Perspektiven und Entwicklungsräume für die Schmerzmedizin!

## Supplementary Information

Entwicklung und Struktur des Fragebogens

## Data Availability

Daten können teilweise auf begründete Anfrage zur Verfügung gestellt werden.

## Footnotes

1 „Der Begriff ‚Schmerzmedizin‘ ist durch ein bio-psycho-soziales Verständnis des Schmerzes begründet und wird von der Deutschen Schmerzgesellschaft e. V. als eine übergreifende Bezeichnung definiert: Alle an der Schmerzforschung, -diagnostik und -therapie beteiligten Fach- und Berufsgruppen (insbesondere Ärzte, Psychologen, Gesundheitsfachberufe u. a.) sind gleichermaßen eingeschlossen.“ – siehe Deutsche Schmerzgesellschaft e. V.

2 Aus Gründen der besseren Lesbarkeit und sprachlichen Vereinfachung wird bei Personenbezeichnungen und personenbezogenen Berufsgruppen im Folgenden die männliche Form verwendet. Entsprechende Begriffe schließen Personen aller Geschlechter und Identitäten mit ein. Die verkürzte Sprachform hat nur redaktionelle Gründe und beinhaltet keine Wertung.
