# Supplementary material for: Professional development and job satisfaction in pain medicine
Source: Schmerz. 2024 Dec 4;40(2):82–95. [Article in German] doi: 10.1007/s00482-024-00851-9 (PMC13004753; doi:10.1007/s00482-024-00851-9)
Supplement: Supplementary file 1 — Entwicklung und Struktur des Fragebogens [file 482_2024_851_MOESM1_ESM.pdf]

Online-Zusatzmaterial zu Methoden

Entwicklung des Fragebogens

Zunächst erfolgten freie Interviews mit Kolleginnen und Kollegen der zu befragenden Berufsgruppen aus dem eigenen Umfeld, zu den Oberthemen „Qualifikation“ und „Berufseinstiege“ und „berufliche Entwicklung“, „Motivation und Arbeitszufriedenheit“. Im Anschluss wurde eine erste Fragenliste mit Antwortmöglichkeiten erstellt, die von der beratenden interdisziplinären und interprofessionellen Gruppe gesichtet und kommentiert wurde (siehe Danksagung). Eine erste Version der Fragen wurde kondensiert und der Fragebogenaufbau inkl. Verzweigungslogik, hinsichtlich auszuschließender Personengruppen, die nicht eingeschlossen werden sollten (=Personen, die bisher nie in einer der oben genannten Einrichtungen schmerzmedizinisch Tätig waren), Personengruppen mit der Notwendigkeit angepasste Frageformulierungen zu erhalten (nicht mehr berufstätige; ehemals in der Schmerzmedizin tätig, aber aktuell nicht; Krankenhausangestellte vs. Niedergelassene und Kombinationen) entworfen. Nach Fertigstellung der Struktur wurde die erste Version des Online-Fragebogens mit dem Programm Unipark (Tivian XI GmbH, Köln) erstellt und anschließend von der Gruppe getestet. In der Testphase konnten hier für jede Frage Kommentare eingefügt werden (diese Funktion wurde nach Abschluss der Testphase deaktiviert). Vor dem Versand erfolgte ein abschließender Projekttest durch das Programm, der eine hohe Anzahl an Testdurchläufen simulierte. Aus der resultierenden Statistik dieser Durchläufe konnten fehlerhafte Einstellungen in den Filterbedingungen ausgeschlossen werden. Bevor die Feldphase des Projektes begann, erfolgte eine Zurücksetzung der Umfrage und alle durch Selbsttests erhobene Daten wurden gelöscht und nicht miteinbezogen.

Fragebogenstruktur

Die endgültige Fragebogenversion umfasste folgende Kapitel die analog in etwa in allen Berufsgruppen abgefragt wurden:

Allgemeine Qualifikation: Hier wurden Abschluss des Medizinstudiums, Facharztqualifikation und Zusatzbezeichnungen [=ZB] erfasst. Der Fokus dabei lag zeitlich darauf, nach wieviel Jahre nach Abschluss des Studiums bzw. nach Facharztqualifikation die Qualifikation zur Schmerzmedizin erfolgte. Ebenso wurde erfasst bei wie vielen Einrichtungen (Kliniken/Abteilungen/Praxen) und im Rahmen welcher Versorgungsformen die schmerzmedizinische Weiterbildung erfolgte (Ambulante Versorgung Hochschul-Ambulanz [=HSA], Ambulante Versorgung MVZ/ Schwerpunkt-/Fachpraxis [=amb. Praxis], Ambulante Versorgung über Ermächtigung [EM], Teilstationäre Assessments [=T-ASS], Teilstationäre Interdisziplinäre Multimodale Schmerztherapie [=S-TK], Stationäre Interdisziplinäre Multimodale Schmerztherapie [=S-IMST], Innerklinische Konsildienste / Schmerzversorgung / Schmerzdienst [=Schmerzdienst], sonstige).

Arbeitserfahrung und schmerzmedizinischer Karriereweg: In diesem Block wurde unabhängig von der Weiterbildung schmerzmedizinische Berufserfahrung erfasst, Zeitpunkt der ersten klinischen schmerzmedizinischen Tätigkeit, Berufsjahre in der Schmerzmedizin ohne relevante Unterbrechungen (max. 6 Monate ohne klinische Tätigkeit in der Schmerzmedizin - Elternzeiten / Mutterschutz / Krankheit zählen hier nicht als Unterbrechung) bzw. kumulierte schmerzmedizinische Berufsjahre. Auch hier wurde die Anzahl der Einrichtungen in denen bereits schmerzmedizinisch gearbeitet wurde erfragt, sowie die Art der Einrichtungen und die Versorgungsformen, in denen die Beantwortenden dort selbst klinisch aktiv tätig waren. Zudem wurde erfasst, ob Arbeitgeberwechsel erfolgten und wie die Befragten an ihre Stelle in der Schmerzmedizin gekommen sind (Initiativbewerbung, abteilungsinterne Rotation / Wechsel, Krankenhausinterne Rotation / Wechsel, öffentliche Ausschreibung, Headhunter, persönliche Ansprache durch die Einrichtung, persönliche Ansprache durch Dritte [hier nicht professionelle Headhunter], Praxisübernahme, sonstiges)

Aktuelle Beschäftigungssituation und Tätigkeit: Dieser Abschnitt erfasste bei denjenigen die zum befragungszeitpunkt klinisch in der schmerzmedizin tätig waren die Charakterisierung der Einrichtung (z.B. eigenständige Klinik, Sektion, Abteilungsbereich, Praxis/ MVZ etc.), der dort

vorhandener Versorgungsformen, der Weiterbildungsbefugnis zur ZB Spez. Schmerztherapie, das Beschäftigungsverhältnis (Teilzeit/Vollzeit, Angestelltenverhältnis/Selbstständigkeit inkl. Mischformen] und die formale Position innerhalb der Einrichtung, Wochenarbeitszeit und deren Anteil für schmerzmedizinische Tätigkeiten, Dienstverpflichtungen. Zudem wurde für die Krankenhäuser Bettenzahl, Träger und Versorgungsstufe und der Status als Universitätskrankenhaus bzw. akademisches Lehrkrankenhaus erfasst. Für die Niederlassung wurde die Grundlage (Kassensitz/persönliche Ermächtigung und Facharztzugehörigkeit des Sitzes/ de Ermächtigung abgefragt.

Motivation und Arbeitszufriedenheit: In diesem Abschnitt sollten durch die Befragten Einflussfaktoren auf einer Nummerischen Ratingskala (NRS 0-10) bewertet werden, inwieweit diese Einflüsse darauf hatten, heute in der Schmerzmedizin tätig zu sein (Kontakt mit dem Bereich während des Studiums, Kontakt mit dem Bereich während Promotion, Persönliche oder familiäre Erfahrung im schmerzmedizinischen Bereich, Fachliches Interesse/ Klinische Tätigkeit als Schmerzmediziner, Einfluss durch Mentor / Vorbild, Erlangen der Zusatzbezeichnung, Beruflichen Position, Verdienst/ Einkommen, Arbeitsbedingungen, Aufstiegsmöglichkeiten, Fort- und Weiterbildungsmöglichkeiten, Umfang und der Länge der Arbeitszeit, Arbeitszeitflexibilität, Vereinbarkeit von Arbeits- und Privatleben, Familienfreundlichkeit, Sicherheit des Beschäftigungsverhältnisses / Einnahmesicherheit, Zusatzverdienstmöglichkeiten, Familienfreundlichkeit, Anerkennung Ihrer Tätigkeit bei Ihren Patienten, Anerkennung Ihrer Tätigkeit bei Kolleginnen/ Kollegen, Anerkennung Ihrer Tätigkeit bei Vorgesetzten). Zudem erfolgte Abfrage der Bewertung (NRS 0-10) der Arbeitsplatzzufriedenheit bzgl. konkreter Aspekte (klinischen Tätigkeit als Schmerzmediziner im Allgemeinen, derzeitige beruflichen Position, derzeitigen Verdienst / Einkommen, derzeitige Arbeitsbedingungen, Aufstiegsmöglichkeiten, Fort- und Weiterbildungsmöglichkeiten, Umfang und der Länge der Arbeitszeit, Arbeitszeitflexibilität, Vereinbarkeit von Arbeits- und Privatleben, Familienfreundlichkeit, Sicherheit des Beschäftigungsverhältnisses / Einnahmesicherheit, Zusatzverdienstmöglichkeiten, Anerkennung der Tätigkeit bei Patienten, bei Kolleginnen/Kollegen und bei Vorgesetzten).

Karriereentwicklung und berufliche Veränderungen: Dieser Abschnitt umfasste die Bewertung anhand der NRS (0-10) des Erreichungsgrades des eigenen Karrierezieles, Wunsch nach weiteren beruflichen Aufstiegsmöglichkeiten in der Schmerzmedizin und die Einschätzung der Aufstiegsmöglichkeit als Schmerzmediziner in der jetzigen Abteilung/Klinik, der weiteren Aufstiegsmöglichkeit als Schmerzmediziner generell auf dem derzeitigen Arbeitsmarkt und der zukünftigen finanzielle Sicherheit durch eine Tätigkeit in der Schmerzmedizin. Abschließend wurde gefragt, ob und welcher Arzt in naher Zukunft eine berufliche Veränderung geplant sei und anhand eines Freitextes, welche drei Aspekte in die Planung / Entscheidung einer beruflichen Veränderung hauptsächlich einwirken würden.

Demographie: Der Abschnitt Demographie erfasste das Alter, das Geschlecht, den akademischen Grad sowie das jährliche Brutto-Einkommen (zur Verbesserung der Antworthäufigkeit gruppiert in Schritten von 20.000 Euro).

Filter und Umgang mit Pflichtfragen

Nach initialer Selektionsfrage und Zuordnung zur Berufsgruppe erfolgte jeweils eine Weiterleitung in einen spezifisch angepassten Fragebogenzweig. Nichtzutreffende Fragen wurde mittels Filter und Ausblendbedingungen nicht angezeigt, um den Fragenumfang auf das notwendige Maß zu reduzieren. Alle Fragen wurden als Pflichtfragen programmiert, sodass keine frei übersprungen werden konnte. Dabei wurde zwischen „weichen“ und „harten“ Pflichtfragen unterschieden. Bei den „weichen“ Pflichtfragen wurde bei Nichtbeantwortung lediglich darauf hingewiesen, dass die Frage wichtig für die Umfrage sei. Bei „harten“ Pflichtfragen war bei Nichtbeantwortung ein Fortführen der Umfrage nicht möglich. „Harte“ Pflichtfragen waren vor allem Filterfragen, die für ein sinnvolles Durchlaufen des Fragebogens bzgl. der Verzweigungslogik essenziell waren.
